# Replacement of fish oil with palm oil: Effects on growth performance, innate immune response, antioxidant capacity and disease resistance in Nile tilapia (*Oreochromis niloticus*)

**DOI:** 10.1371/journal.pone.0196100

**Published:** 2018-04-25

**Authors:** Christian Larbi Ayisi, Jinliang Zhao, Jun-Wei Wu

**Affiliations:** Key Laboratory of Freshwater Fishery Germplasm Resources, Ministry of Agriculture, Shanghai Ocean University, Shanghai, P. R. China; University of Illinois, UNITED STATES

## Abstract

This study was conducted to investigate the effects of replacing dietary fish oil (FO) with palm oil (PO) in juvenile Nile tilapia, *Oreochromis niloticus* (9.34± 0.02g initial weight) with emphasis on growth performance, digestive enzyme activities as well as serum biochemical parameters. Also, lysozyme activity (LYZ), respiratory burst (RB), superoxide dismutase (SOD), catalase (CAT) and resistance to *Streptococcus iniae* were investigated. Fish were stocked in 15 rectangular fiber glass tanks (150× 60× 40 cm) at 40 fish per tank with water maintained at 210 litres. Fish were fed five isonitrogenous (33% crude protein) and isolipidic (10% lipid) diets with PO included at 0% (0% PO), 25% (25% PO), 50% (50%PO), 75% (75% PO) and 100% (100% PO) for 8 weeks. The findings demonstrated that growth, and feed utilization was not compromised when PO was used in place of FO either partially or totally. Except for protease activity which was not significantly altered, lipase and amylase activities were significantly altered when FO was replaced with PO. There were no significant differences among treatments for CAT, SOD and LYZ. Serum malondialdehyde (MDA) in fish fed 100% PO was significantly lower than all other groups whiles total antioxidant capacity (TAC) of fish fed 0% PO was significantly higher than all other groups. Fish fed 0% PO, 25% PO and 50% PO had glutathione reductase (GR) significantly higher than fish fed 75% PO and 100% PO. RB in fish fed 0% PO were significantly lower than fish fed 75% PO and 100% PO. Also, fish fed 0% PO had significantly lower total protein (TP) compared with groups fed 50% PO and 75% PO. Fish fed diets with PO had similar resistance ability to *Streptococcus iniae* as those fed diets with FO. However, the liver function was likely to be compromised due to the increase in aspartate amino transferase (AST) and alkaline phosphatas (ALP) along increasing PO inclusion levels. AST, total protein, triacylglycerol (TAG) and low-density lipoprotein cholesterol (LDL-C) were significantly higher (p<0.05) in groups fed higher levels of PO. This study therefore concludes that feeding tilapia fingerlings with diets containing PO affects antioxidant and innate immune parameters negatively due to the reduction in LYS, TAC, GR, MDA, CAT, SOD and GSHpx.

## Introduction

Dietary lipid cannot be replaced by other nutrients because it plays important biological functions, and serve as a major source of essential fatty acid and provide energy [1]. Fish oil has traditionally been used as the major lipid component in fish feed [2] to provide essential polyunsaturated fatty acid (PUFA), especially highly unsaturated fatty acid (HUFA) [3]. However, limited output cannot meet the increasing demand of aqua feed industry [4].

Vegetable oils (VO) are potential and suitable candidates to replace fish oils in aquaculture feeds [5] because they have relatively considerable output, acceptable price, relatively low organic contaminant status and relatively high content of unsaturated fatty acids. Addition of VO in diets have resulted in a compromise in non-specific immunity parameters of fish such as large yellow croaker [6] and Japanese sea bass [7].

Lysozyme is a lytic protein that is important in the non-specific defense system. Serum lysozyme activity are relevant since it provides a defense line in preventing proliferation and colonization of pathogenic microbes as a consequence ensuring in the diminution of disease [8]. Serum proteins are the most important compounds in the serum, with albumin and globulin being the major serum proteins [9] and are used to predict the general well-being of organisms including fish.

Superoxide dismutase (SOD) and catalase (CAT) are some essential antioxidant enzymes in fish, they are responsible for the breakdown and activation of the main metabolically produced endogenous radicals, hydrogen peroxide and superoxide [10], thus, they play important role in the innate immune system [11]. Malondialdehyde (MDA) is an indicator commonly used to evaluate lipid peroxydation and carbonyl proteins ([12] as the level of MDA indicates that higher peroxidation leads to cell degradation [13].

To our knowledge, limited information is available on the effects of replacing FO with PO on antioxidant capacity, immune response, digestive enzyme activities and resistance to *S*. *iniae* in tilapia. This study will give a fair idea concerning how replacing FO with PO could impact the health of *O*. *niloticus*. The objective of the present study is therefore to determine the effects of replacing FO with PO on growth performance, antioxidant capacity, immune response, serum metabolites, digestive enzyme activities and response to *S*. *iniae* in juvenile Nile tilapia.

## Materials and methods

### Feed and feeding trial

Five diets; isonitrogenous (33% crude protein) and isolipidic (10% crude lipid) were formulated for this study with ingredients supplied by Nonghao Feed Company (Shanghai, China). The differences in diets are with respect to fish oil and palm composition (Table 1). Fish oil (FO) was replaced with Palm oil (PO) at 0%, 25%, 50%, 75% and 100% and designated as 0% PO, 25% PO, 50% PO, 75% PO and 100% PO respectively. Protein sources were fish meal, rapeseed meal and soybean meal with fish oil and palm oil being lipid sources. Dry ingredients were mixed first in a progressive manner after which they were mixed with fish oil, palm oil and water into dough using a Hobart mixer. Meat mincer with a 1 mm die was then used to produce pellets out of the dough. All pellets were dried, broken into smaller pieces and finally stored at -20 C until time of feeding. Table 2 shows the analyzed fatty acid composition of the experimental diets. Gross energy estimated according to [14] using the values of 23.6, 39.5 and 17.2 kJ/g for crude protein, lipid and total carbohydrates respectively.

**Table 1:** Formulation and proximate composition of experimental diets (g/100 g in dry matter).

Dietary Palm oil (PO) replacement level^1^					
Ingredient	0% PO	25% PO	50% PO	75% PO	100% PO
Fish meal*	6.00	6.00	6.00	6.00	6.00
Soybean meal*	30.00	30.00	30.00	30.00	30.00
Wheat meal*	22.50	22.50	22.50	22.50	22.50
Rapeseed meal*	30.00	30.00	30.00	30.00	30.00
Fish oil*	6.00	4.50	3.00	1.50	0.00
Palm oil*	0.00	1.50	3.00	4.50	6.00
Soybean phospholipid*	2.50	2.50	2.50	2.50	2.50
Mineral mix**	0.55	0.55	0.55	0.55	0.55
Vitamin mix***	0.40	0.40	0.40	0.40	0.40
Ca(H_2_PO_4_)	1.50	1.50	1.50	1.50	1.50
Choline Chloride	0.50	0.50	0.50	0.50	0.50
Inositol	0.05	0.05	0.05	0.05	0.05
Total	100	100	100	100	100
Proximate composition (%)					
Moisture	10.30	10.53	10.82	10.70	10.50
Protein	33.18	33.15	33.09	33.23	33.17
Lipid	9.82	9.81	9.87	9.88	9.85
Ash	5.25	5.60	5.53	5.40	5.50
Carbohydrate^****^	41.45	40.91	40.69	40.79	40.98
Estimated energy (kJ/g)	1883.88	1873.49	1870.66	1876.08	1876.74

^1^Experimental diet abbreviations: 0% PO (100% FO, 0% PO), 25% PO (75% FO, 25% PO), 50% PO (50% FO, 50% PO), 75% PO (25% FO, 75% PO), 100% PO (0% FO, 100% PO)

*Fish meal, Soybean meal, Wheat meal, Soybean phospholipase, Palm oil, Vitamin premix, Mineral mix and Ca (H_2_PO_4_) were supplied by Nonghao Feed Company (Shanghai, China).

**Mineral mix (mg kg^-1^ dry diet): Cu (CuSO_4_), 2.0; Zn (ZnSO_4_), 34.4; Mn (MnSO_4_), 6.2; Fe (FeSO_4_), 21.1; I (Ca (IO_3_)_2_), 1.63; Se (Na_2_SeO_3_), 0.18; Co (CoCl_2_), 0.24; Mg (MgSO_4_.H_2_O), 52.7.

*** Vitamin premix (IU or mg kg^-1^ diet): vitamin A, 16000 IU; vitamin D, 8000 IU; vitamin K, 14.72; thiamin, 17.8; riboflavin, 48; pyridoxine, 29.52; cynocobalamine, 0.24, tocopherols acetate, 160; ascorbic acid (35%), 800; niacinamide, 79.2; calcium-D- pantothenate,73.6; folic acid, 6.4; biotin, 0.64 L-carnitine, 100.

^****^Carbohydrates % = 100- (crude protein % + crude lipid % + moisture % + ash %).

**Table 2:** Main fatty acid composition (% of total fatty acids) of experimental diets.

Fatty acid(s)	Dietary Palm oil (PO) replacement level^1^				
	0% PO	25% PO	50% PO	75% PO	100% PO
12:0	0.15±0.00	0.14±0.01	0.12±0.00	0.12±0.01	0.11±0.00
14:0	5.54±0.21	4.52±0.04	3.20±0.05	2.25±0.09	1.27±0.10
16:0	23.88±0.49	25.90±0.08	27.00±0.39	28.06±0.37	29.19±0.27
18:0	5.31±0.04	5.76±0.06	5.43±0.12	5.30±0.13	5.15±0.05
Total SFA	34.88±0.46	36.32±0.83	35.75±0.19	35.73±0.33	37.72±0.72
16:1(n-7)	6.02±0.34	5.23±0.59	3.15±0.04	2.06±0.15	0.89±0.01
18:1(n-9)	23.56±0.68	25.42±0.09	28.40±0.16	31.16±0.20	33.77±0.16
Total MUFA	29.58±0.85	30.65±0.47	31.55±0.77	33.22±0.43	34.66±0.32
18:2(n-6)	20.65±0.77	21.57±0.05	23.07±0.22	23.62±0.32	24.33±0.03
20:4(n-6) ARA	0.56±0.05	0.47±0.03	0.41±0.00	0.25±0.05	0.20±0.02
Total n-6	21.21±0.22	22.04±0.89	23.48±0.33	23.87±0.18	24.53±0.63
18:3(n-3)	5.52±0.23	3.96±0.15	3.98±0.04	3.79±0.09	3.63±0.03
18:4(n-3)	0.32±0.01	0.33±0.00	0.32±0.01	0.30±0.00	0.29±0.01
20:5(n-3) EPA	4.25±0.14	3.19±0.01	2.20±0.06	1.41±0.04	0.62±0.00
22:6(n-3) DHA	5.66±0.35	4.15±0.04	2.85±0.16	1.69±0.02	0.58±0.02
Total n-3	15.75±0.19	11.63±0.57	9.35±0.04	7.19±0.39	5.12±0.31
DHA/EPA	1.33±0.05	1.30±0.21	1.29±0.33	1.19±0.43	0.93±0.05
Total PUFA	34.96±0.18	33.67±0.34	32.83±0.18	31.06±0.09	29.65±0.14
Total SFA/ total PUFA	0.87	1.07	1.08	1.15	1.20
n-3:n-6	0.74	0.52	0.39	0.30	0.20

^1^ Experimental diet abbreviations: 0% PO (100% FO, 0% PO), 25% PO (75% FO, 25% PO), 50% PO (50% FO, 50% PO), 75% PO (25% FO, 75% PO), 100% PO (0% FO, 100% PO)

ARA = Arachidonic acid; EPA = Eicosapentanoic acid; DHA = Decosahexanoic acid; SFA = saturated fatty acids; MUFA = mono unsaturated fatty acid; PUFA = polyunsaturated fatty acid.

### Ethics statement

The present study was performed in strict accordance with the Standard Operation Procedures (SOPs) of the Guide for the Use of Experimental Animals of Shanghai Ocean University. All animal care and use procedures were approved by the Institutional Animal Care and Use Committee of Shanghai Ocean University. Fish were anesthetized with excess tricaine methane sulfonate (MS-222 at 200mg/L) before sampling in order to reduce suffering and stress.

### Experimental procedures

Mixed sex Nile tilapia fingerlings were obtained from Tilapia Germplasm Station of Shanghai Ocean University, China. Fish were sent to Shanghai Ocean University’s aquarium facility where they were fed twice a day to apparent satiation on commercial diets (30% crude protein) purchased from Shanghai Jin Yuan Trade. Twenty fours before trial, fish were starved, distributed into their respective tanks (fiber glass tanks of 150× 60× 40 cm) at a stocking density of 40 fish per tank. Each group was then fed their corresponding diet for eight week, two times a day at 08:00 and 16:00 to apparent satiation. Following standard methods [15], nitrite-N and ammonia-N were measured once a week whiles YSI 556 instrument (YSI, Yellow Springs, Ohio) was used to measure water temperature, dissolved oxygen and pH every day. The initial weight of fish was 9.34±0.02.

### Sample collection and growth performance

Feed was withheld from fish 24 hours before sampling. Fifteen fish were then sampled from each treatment (five from each tank), euthanized with an overdose of tricaine methane sulfonate (MS-222 at 200mg/L) as previously described by [16]. Weight and length were then measured in grams and centimeters (cm) respectively. Tissues (liver and muscle) were sampled and stored at -80°C for measurement of proximate composition. Growth performance and feed efficiency were measured using indicators shown below:

(1) Total weight gain (WG)
  WG=FW(g)−IW(g)
  FW and IW represent final weight and initial weight, respectively.
(2) Feed intake (FI) is the total feed consumed (g) during the 56 days trial.
(3) Feed conversion ratio (FCR)
  FCR=FI(g)/WG(g)
(4) Survival (S)
  $$\text{S}=\left(\frac{\text{TF}}{\text{TFT}}\right)\text{*}100$$
  TF and TFT represent total number of fish stocked and total number of fish at end of trial, respectively.
(5) Condition factor (C)
  $$\text{C}=\left[\frac{\text{BW}}{\left(\text{TL}\right)3}\right]\text{*}100$$
  BW and TL represent body weight (g) and length (cm) respectively.

### Fatty acid analysis of experimental diets, muscle and liver

Analysis of fatty acids in diets, muscle and liver were performed as previously described in [17]. Feed as well as muscle and liver were grinded into fine powder. Chloroform-methanol in the ratio of 2:1 (V/V) was used to extract total lipids according to the [18] method. 0.4 M KOH-methanol was used to prepare fatty acid methyl esters by transesterification. Individual fatty acids were detected by gas chromatograph (GC-7890A) with methyl heneicosanoate (C21:0) as an internal standard. Peak times of each sample were compared to that of the manufacturer to identify individual fatty acids. The percentages of the individual fatty acids were determined using peak areas. All measurements were performed in triplicates and the fatty acids content expressed as % total FA.

### Digestive enzyme activity

Digestive tract of five fish per tank that were previously bled were excised and divided into three portions: anterior, middle and posterior. The posterior intestine was distinguished from the mid intestine by increased diameter, darker mucosa, and annular rings. The remaining part was divided into two identical parts to obtain the anterior and mid portions. The samples were then frozen in liquid nitrogen and stored at -80 until measurement of enzyme activity. All enzyme activities were measured as previously described by [19].

### Serum metabolites

Fifteen fish were sampled from each treatment (five from a tank), anesthetized with excess amount of tricaine methane sulfonate (MS-222 at 200mg/L and blood drawn from the caudal vein using a 1-mL syringe with a 22-gauge × 3.8-cm (11/2 in) needle. Sampled blood was then transferred into a 1.5-mL micro centrifuge tubes, centrifuged at 3500 rpm for 15 min. The supernatant were then transferred to new tubes and stored at −20°C for the subsequent analyses. Contents of alanine amino transferase (ALT), aspartate amino transferase (AST), alkaline phosphate (ALP) high density lipoprotein-cholesterol (HDL-C), low density lipoprotein-cholesterol (LDL-C), total cholesterol (TC) and triacylglycerol (TAG), were analyzed using a biochemical analyzer (Mindary Chemistry Analyzer BS-200, Shenzhen, China) as previously described by [16]. The kits used were supplied by Jiancheng Biological Limited (Nanjing, China). In brief, 0.5–1.0 ml of serum were pipetted into 1.5ml eppendorf tubes and inserted into the biochemical analyzer. The analyzer was calibrated according to the manufactures instruction and results read on attached computer.

### Serum total protein, serum lysozyme (LYZ) and respiratory burst (RB) activity assay

Serum total protein content was analyzed using a biochemical analyzer (Mindary Chemistry Analyzer BS-200, Shenzhen, China) as described above. Turbidimetric assay method according to [20] was used to measure LYZ content whiles nitro-blue tetrazolium (NBT; Sigma, USA) assay described by [21] with some modifications by [22] was used to measure respiratory burst activity. The optical density of the supernatant was measured at 540 nm using dimethylformanide as the blank.

### Measurement of antioxidant activities

Commercial kit from Nanjing Jiancheng Biotech Co. (Nanjing, China) was purchased for the analysis of SOD activity Analysis was performed as described by the manufacturer. The amount of superoxide dismutase needed to inhibit the reduction of nitro blue tetrazolium by 50% defined one unit of SOD activity and was expressed as Aspartate aminotransferase (GOT) and alanine aminotransferase (GPT) activities were measured as previously described by [23] whiles Catalase (CAT) was determined by the decomposition of hydrogen peroxide as previously described by [24]. Thiobarbituric acid reactive substances (TBARS) was used to determine Malondialdehyde (MDA) content as described by [25] using an MDA assay kit (Jiancheng Biotech. Co. Nanjing, China). MDA undergoes a condensation reaction in the presence of thiobarbituric acid and generates a red product with a maximum absorption peak at 532 nm.

Commercial kits were purchased from Nanjing Jiancheng Bioengineering Institute (Jiangsu, China) for analysis of Glutathione peroxidase (GSH-Px), glutathione reductase (GR), and total antioxidant capacity (TAC) activities in serum using a spectrophotometer (UV-2100, Shanghai Jinhua Technology Instrument Co., Ltd., Shanghai, China) at 412, 340, and 520 nm, respectively. 0.01 increment of absorbance of the reaction system caused by serum per milliliter reacting at 37 C for 1 min defined one unit of TAC. The amount of enzyme that reduces the concentration of GSH in the reaction system at 1mmol l^-1^ per min defined one unit of GSH-Px whiles a unit of GR activity was defined as the amount of enzyme depleting 1 mmol NADPH in 1 min [26]. Using 1-chloro-2, 4-dinitrobenzene as substrate Glutathione s-transferase (GST) was measured by absorbance at 340 nm as previously described in [27].

### Bacterial challenge

Frozen stock-culture of *Streptococcus iniae* (*S*. *iniae*—ARS-98-60) was obtained from National Pathogen Collection Center for Aquatic Animals (Shanghai Ocean University). The bacterium was cultured following procedures described previously by [28]. In order to ascertain the lethal dose, 96 h LD50 was determined by intraperitoneal injection using graded doses of *S*. *iniae* (10^4^ 10^5^, 10^6^, 10^7^ and 10^8^ CFU per fish). The results showed that the 96 h LD50 was 10^5^ CFU per fish.

Ten fish from each tank (30 per treatment) were randomly sampled after the 56 days feeding trial for the bacterial challenge. All fish were kept in a 57-L aquaria containing 50 L water. Water temperature was maintained at 28–29 C. Fish were injected with a dose of 0.1mL of 1 × 10^6^ cfu/mL of *S*. *iniae* (10 × 10^5^ cfu/fish) intra-peritoneal (IP) using tuberculin syringe. The fish were fed their respective diets for 14 days whiles they were monitored and mortality recorded twice daily.

### Statistical analysis

Data was analyzed using both univariate and multivariate GLM and no significant interactions were observed so we only report one-way ANOVA. Tukey’s multiple tests were used to compare means of all treatments. Significant differences was tested at *P<0*.05. Normality of data and homogeneity of variance were tested using Shapriro-Wilk normality test and Bartlett's test respectively. Graph Pad Prism (V.5.03) was used to perform all analysis data presented as mean ± standard error of the mean (SEM).

## Results

### Survival, growth performance and feed utilization

Survival, growth performance, feed utilization and morphometric index are shown in Table 3. Fish survival was 100% in all groups during the experimental treatments. The final body weight (FBW) and body weight gain (BWG) in fish fed 50% PO diet was significantly higher than in 25% PO but was non-significantly different from other groups (*P<0*.*05*). However there was no difference among groups for condition factor (K) among the different groups (*P*>0.05). There was no difference among groups with respect to feed intake and ranged between 76.27 and 77.19.

**Table 3:** Growth performance and nutrient utilization of *Oreochromis niloticus* fed experimental diets.

Dietary Palm oil (PO) replacement level (%)^1^												
Parameters	0% PO	CV	25% PO	CV	50% PO	CV	75% PO	CV	100% PO	*CV*	*P value*	*r*
Initial length(cm)	6.73±0.08	7.84	6.75±0.06	6.49	6.71±0.07	6.61	6.72±0.06	6.14	6.73±0.05	4.95	0.995	0.0316
Initial weight (g)	9.35±0.05	1.02	9.29±0.03	0.66	9.30±0.05	1.01	9.38±0.05	0.98	9.42±0.03	0.66	0.354	0.5761
**Final weight (g)**	66.34±1.03^a^^b^	2.62	62.48±0.91^a^	3.41	69.85±2.19^b^	5.44	64.42±1.15^a^^b^	2.01	65.05±1.11^a^^b^	3.10	0.032	0.7867
**WG**^**2**^	56.98±0.96^a^^b^	2.95	53.18±1.26^a^	4.11	60.54±2.14^b^	6.12	55.04±0.71^a^^b^	2.26	55.63±1.17^a^^b^	3.65	0.030	0.7914
FI (g)^3^	76.74±0.21	0.49	76.27±0.26	0.61	77.64±0.26	0.59	76.69±0.39	0.88	77.19±0.35	0.79	0.076	0.7348
FCR^4^	1.34±0.02	2.59	1.43±0.03	4.81	1.28±0.03	5.38	1.39±0.02	2.91	1.38±0.03	4.32	0.070	0.7404
SR (%) ^5^	100.00±0.00	0.00	100.00±0.00	0.00	100.00±0.00	0.00	100.00±0.00	0.00	100.00±0.00	0.00	N/A	N/A
C^6^	2.14 ±0.06	5.65	1.99 ±0.06	5.49	2.16 ±0.06	4.82	2.07 ±0.06	5.33	2.02 ±0.04	3.82	0.278	0.6102

^1^ Experimental diet abbreviations: 0% PO (100% FO, 0% PO), 25% PO (75% FO, 25% PO), 50% PO (50% FO, 50% PO), 75% PO (25% FO, 75% PO), 100% PO (0% FO, 100% PO).

^2^Weight Gain;

^3^Feed Intake;

^4^Feed Conversion Ratio;

^5^Survival Rate;

^6^Condition Factor; Values are mean ± SEM (n = 3). Different superscripts indicate significant difference among different treatments (*P*<0.05).

Superscript a is significantly less than

b. Parameters highlighted have significant differences among groups. CV is coefficient of variation and r is correlation

### Lipid and fatty acids composition

Effects of replacing fish oil with palm oil on fatty acid composition of *O*.niloticus has been reported and discussed in our previous study [17]. A summary of lipid content as well as fatty acid composition of liver and muscle is presented in Tables 4 and 5 respectively. In brief, as PO inclusion levels increased, liver total saturated fatty acids (SFA), mono unsaturated fatty acids (MUFA) and 18:2n-6 (LA) increased. EPA, DHA and total poly unsaturated fatty acids (PUFA) decreased with increasing PO inclusion. In the muscle, increasing PO levels resulted in an increase of EPA, DHA, total n-3 PUFA, total PUFA, n-3: n-6 ratio and 20:2n-6. There was no difference in the lipid content of liver and muscle. The lipid content in the liver and muscle ranged between 7.48 to 9.06 and 9.15 to 9.48 respectively.

**Table 4:** Lipid and fatty acid composition (% of total fatty acids) in liver of *Oreochromis niloticus* juvenile fed experimental diets.

	Dietary Palm oil (PO) replacement level (%)^1^											
Fatty acid(s)	0% PO	CV	25% PO	CV	50% PO	CV	75% PO	CV	100% PO	CV	p-value	r
Lipid	7.48±0.86	17.64	7.63±0.43	13.98	8.46±0.59	17.13	8.61±0.78	22.46	9.06±0.41	11.15	0.1065	0.5043
**18:2(n-6)**	9.64±0.00^a^	0.07	10.60±0.14^b^	1.78	12.04±0.39^b^	5.70	14.13±0.17^c^	1.77	14.56±0.67^c^	7.98	0.0001	0.9637
18:3(n-3)	0.91±0.02	4.42	0.98±0.01	1.88	0.96±0.15	27.80	1.00±0.49	27.41	1.15±0.00	1.13	0.8624	0.3672
**20:4(n-3)**	0.96±0.01^b^	5.12	0.77±0.15^b^	20.00	0.91±0.01^b^	10.99	0.38±0.00^a^	3.01	0.29±0.01^a^	8.14	0.0017	0.9666
20:4(n-6)	0.98±0.02	4.53	0.68±0.03	16.54	0.72±0.01	4.34	1.00±0.21	14.32	0.87±0.01	2.72	0.4513	0.4513
**20:5(n-3)**	1.06±0.02^b^	4.80	0.58±0.15^a^^b^	5.57	0.20±0.03^a^	8.41	0.27±0.03^a^	18.7	0.13±0.00^a^	8.65	0.0014	0.9304
**22:6(n-3)**	6.35±0.18^c^	5.06	5.85±0.31^c^	7.57	4.33±0.09^b^	3.70	1.47±0.19^a^	18.84	0.85±0.02^a^	5.34	0.0001	0.9959
**Σ SFA**	37.18±0.46^a^	0.05	37.76±0.83^b^	0.61	38.25±0.19^c^	0.05	39.57±0.33^d^	1.46	39.70±0.72^e^	1.28	0.0056	0.9593
**Σ MUFA**	38.98±0.85^a^	2.57	39.79±0.47^b^	2.06	42.14±0.77^c^	0.86	42.42±0.43^d^	0.95	43.55±0.32^e^	1.87	0.0001	0.9456
**Total PUFA**	23.27±0.20^c^	1.58	21.48±0.46^b^^c^	2.81	20.86±0.43^b^	3.63	19.17±0.43^a^^b^	3.19	18.50±0.70^a^	6.59	0.0007	0.9419
**Σ n-3**	10.16±0.22^e^	3.12	8.81±0.89^d^	2.44	6.88±0.33	2.94	3.65±0.18^b^	11.57	2.77±0.63^a^	3.30	0.0001	0.9975
**Σ n-6**	13.10±0.02^a^^b^	0.31	12.66±0.21^a^	2.62	13.98±0.36^a^^b^^c^	4.52	15.52±0.13^b^^c^	1.22	15.73±0.68^c^	7.59	0.0033	0.9129
**n-3:n-6**	0.77±0.04^c^	7.90	0.69±0.02^c^	6.26	0.49±0.01^b^	6.12	0.24±0.01^a^	9.49	0.18±0.00^a^	3.09	0.0058	0.9915

^1^Experimental diet abbreviations: 0% PO (100% FO, 0% PO), 25% PO (75% FO, 25% PO), 50% PO (50% FO, 50% PO), 75% PO (25% FO, 75% PO), 100% PO (0% FO, 100% PO). ΣSFA’s includes C12:0, C14:0, C16:0, C18:0 and C20:0; Σ MUFAs includes C16:1n-7, C16:1n-9, C18:1n-7 and C18:1n-9. Values are mean ± SEM (n = 15). Different superscripts indicate significant difference among different treatments (*P*<0.05).

Superscript a is significantly less than

b. Superscript b is significantly less than

c. Superscript c is significantly less than

d. Superscript d is significantly less than

e. Parameters highlighted have significant differences among groups. CV is coefficient of variation and r is correlation.

**Table 5:** Lipid and fatty acid composition (% of total fatty acids) in muscle of *Oreochromis niloticus* juvenile fed experimental diets.

Dietary Palm oil (PO) replacement level (%)^1^												
Fatty acids	0% PO	CV	25% PO	CV	50% PO	CV	75% PO	CV	100% PO	CV	p-value	r
Lipid	9.48±0.12	15.8	9.82±0.38	1.94	9.54±0.05	1.09	9.89±0.74	2.71	9.15±0.08	7.86	0.6681	0.3714
**18:2(n-6)**	10.59±0.43^a^^b^	7.04	12.48±0.61^b^	8.50	10.36±0.19^a^	3.19	10.30±0.21^a^	3.66	10.99±0.43^a^^b^	6.94	0.0021	0.8120
**18:3(n-3)**	0.50±0.04^a^	14.00	0.81±0.00^a^^b^	0.72	1.06±0.11^b^^c^	18.58	1.22±±0.03^c^	4.35	1.16±0.09^c^	14.20	0.0041	0.9370
**20:4(n-3)**	0.54±0.01^a^^b^	4.60	0.52±0.03^a^	10.46	0.58±0.02^a^^b^	8.58	0.58±0.02^a^^b^	5.92	0.65±0.02^b^	5.55	0.0229	0.7805
**20:4(n-6)**	1.62±0.13^d^	13.88	1.2±0.07^c^	10.83	0.68±0.01^b^	4.41	0.50±0.04^a^^b^	15.95	0.26±0.00^a^	4.00	0.0027	0.9803
**20:5(n-3)**	0.97±0.03^c^	6.0	0.84±0.07^b^^c^	14.67	0.77±0.10^a^^b^^c^	22.63	0.56±0.05^a^^b^	15.57	0.47±0.02^a^	7.42	0.0018	0.9026
**22:6(n-3)**	4.80±0.07^c^	2.76	4.32±0.03^c^	1.39	4.09±0.26^c^	11.13	2.46±0.18^b^	13.90	1.57±0.06^a^	6.99	0.0006	0.9845
**ΣSFA’s**	35.23±0.13^a^	0.65	35.60±0.34^a^	1.67	40.06±0.34^b^	1.49	40.68±0.35^b^	1.53	40.75±0.19^b^	0.73	0.0345	0.9869
**Σ MUFAs**	43.84±0.12	0.50	43.29±0.05	0.23	43.62±0.89	3.57	45.02±0.25	1.00	44.18±0.40	1.57	0.6738	0.6738
**Total PUFAs**	20.63±0.38^c^^d^	3.24	21.63±0.58^d^	4.65	18.69±0.43^b^^c^	4.01	16.62±0.50^a^^b^	5.23	15.80±0.50^a^	5.54	0.0001	0.9559
**Σ n-3**	7.59±0.03^c^	0.90	7.17±0.13^c^	3.21	7.06±0.36^c^	8.63	5.37±0.08^b^	5.73	4.18±0.07^a^	4.50	0.0002	0.9808
**Σ n-6**	13.04±0.38^b^	5.11	14.46±0.70^c^	8.47	11.62±0.15^a^^b^	2.31	11.25±0.24^a^^b^	3.84	11.61±0.43^a^	6.65	0.0401	0.8910
**n-3:n-6**	0.58±0.01^c^^d^	2.99	0.49±0.03^b^^c^	3.10	0.60±0.02^d^	5.82	0.47±0.01^b^	4.84	0.36±0.00^a^	2.09	0.0366	0.9479

^1^ Experimental diet abbreviations: 0% PO (100% FO, 0% PO), 25% PO (75% FO, 25% PO), 50% PO (50% FO, 50% PO), 75% PO (25% FO, 75% PO), 100% PO (0% FO, 100% PO). ΣSFA’s includes C12:0, C14:0, C16:0, C18:0 and C20:0; Σ MUFAs includes C16:1n-7, C16:1n-9, C18:1n-7 and C18:1n-9. Values are mean ± SEM (n = 15). Different superscripts indicate significant difference among different treatments (*P*<0.05).

Superscript a is significantly less than

b. Superscript b is significantly less than

c. Superscript c is significantly less than

d. Parameters highlighted have significant differences among groups. CV is coefficient of variation and r is correlation

### Digestive enzyme activity

Replacing dietary FO with PO resulted in different response with respect to the analyzed digestive enzyme activities (Table 6). In this study, inclusion of dietary PO resulted in a significant difference (p<0.05) in amylase and lipase of all digestive regions. In anterior intestine (AI), middle intestine (MI) and posterior intestine (PI), there was a trend of increasing lipase activity along increasing PO levels. Fish fed diets containing 50% PO recorded the highest amylase activity in AI, MI and DI. There was no difference among different groups in protease activity of AI, MI and PI (*P*>0.05). However, the highest activity in AI, MI and PI were recorded in fish fed 50% PO.

**Table 6:** Specific digestive enzyme activities (mU g^-1^protein) in anterior intestine (AI), mid intestine (MI) and posterior intestine (PI) of *Oreochromis niloticus* fed experimental diets.

Enzymes	Sections	Dietary Palm oil (PO) replacement level (%)^1^											
		0% PO	CV	25% PO	CV	50% PO	CV	75% PO	CV	100% PO	CV	p-value	r
	AI	0.31±0.007^b^	6.93	0.25±0.001^a^	1.30	0.32±0.005^b^	4.95	0.25±0.004^a^	5.44	0.26±0.001^a^	3.25	0.0001	0.8619
**Amylase**	MI	0.28±0.006^b^	15.33	0.24±0.006^a^	7.75	0.32±0.005^b^	5.31	0.28±0.002^a^^b^	2.94	0.24±0.002^a^	3.00	0.0001	0.5181
	PI	0.29±0.004^c^	4.91	0.22±0.002^a^	2.69	0.30±0.000^c^	0.94	0.29±0.008^c^	8.54	0.25±0.003^b^	3.60	0.0001	0.9149
	AI	158.10±2.56^a^	20.75	257.67±2.28b	12.23	428.89±2.16^c^	3.31	419.57±13.02^c^	2.89	613.66±2.54^d^	2.48	0.0001	0.9928
**Lipase**	MI	133.86±3.08^a^	32.24	185.78±3.66^a^	9.16	444.05±2.81^b^	1.75	424.20±2.10^b^	1.01	588.49±4.73^b^	0.88	0.0001	0.9577
	PI	124.88±7.28^a^	7.79	157.65±10.19^a^	19.72	392.71±12.32^b^	5.76	396.67±7.99^b^	3.42	690.52±10.82^c^	1.39	0.0001	0.9975
	AI	459.6±52.52	25.56	305.0±29.39	21.55	460.7±55.96	27.16	359.69±69.07	42.96	381.1±30.32	17.79	0.1665	0.5155
Protease	MI	448.4±69.99	38.23	378.1±40.77	26.41	487.7±54.66	27.46	419.0±44.84	26.21	421.8±62.03	36.02	0.7140	0.2798
	PI	428.8±79.36	20.48	354.4±45.69	31.58	459.2±69.84	37.26	385.2±75.25	47.84	391.4±25.30	15.83	0.7902	0.2518

^1^ Experimental diet abbreviations: 0% PO (100% FO, 0% PO), 25% PO (75% FO, 25% PO), 50% PO (50% FO, 50% PO), 75% PO (25% FO, 75% PO), 100% PO (0% FO, 100% PO). AI = anterior intestine; MI = middle intestine; PI = posterior intestine. Values are mean ± SEM (n = 15). Different superscripts indicate significant difference among different treatments (*P*<0.05).

Superscript a is significantly less than

b. Superscript b is significantly less than

c. Superscript c is less than superscript

d. Parameters highlighted have significant differences among groups. CV is coefficient of variation and r is correlation

### Serum biochemical indices

Serum biochemical indices analyzed are summarized in Table 7. There were significant differences among treatment groups for aspartate aminotransferase (AST), triacylglycerol (TAG), alkaline phosphate (ALP), HDL-C, LDL-C and HDL-C/LDL-C ratio (*P<*0.05). On the contrary, there were no differences among treatment groups for total cholesterol (TC), Glutamic-pyruvic transaminase (GPT) and Glutamic- oxalacetic transaminase (GOT). HDL-C and HDL-C/LDL-C ratio significantly decreased with increasing dietary PO inclusion whiles ALP, AST, and LDL-C and TAG significantly increased with increasing dietary PO inclusion. Generally, GOT and GPT increased with increasing PO inclusion levels but were not significant.

**Table 7:** Serum biochemical indices of *Oreochromis niloticus* fed experimental diets.

	Dietary Palm oil (PO) replacement level (%)^1^											
Parameters	0% PO	CV	25% PO	CV	50% PO	CV	75% PO	CV	100% PO	CV	*P*- value	*r*
TC (mmol L^-1^)^2^	3.51±0.16	10.68	3.52±0.07	4.59	3.75±0.24	14.34	3.89±0.30	17.56	3.99±0.05	3.33	0.335	0.4415
**TAG (mmol L**^**-1**^**)**^**3**^	3.26±0.23^a^	16.19	4.03±0.03^a^^b^	1.67	4.76±0.17^b^^c^	8.20	5.16±0.43^b^^c^	19.03	5.57±0.40^c^	16.32	0.000	0.8089
HDL-C (mmol L^-1^)^4^	2.32±0.33	19.07	2.30±0.27	5.21	2.40±0.13	12.22	2.19±0.15	15.54	1.60±0.07	5.63	0.253	0.6898
**LDL-C (mmol L**^**-1**^**)**^**5**^	0.27±0.02^a^	18.09	0.30±0.2^a^^b^	23.09	0.32±0.02^a^^b^	20.04	0.42±0.04^b^	22.32	0.43±0.01^b^	4.65	0.007	0.7158
**HDL-C/LDL-C**	8.41±1.30^b^	13.69	8.28±1.88^b^	20.73	7.72±1.06^b^	20.90	5.42±0.69^a^^b^	20.45	3.78±0.07^a^	3.35	0.008	0.7340
**AST (UL**^**-1**^**)**^**6**^	17.50±1.83^a^	13.40	20.86±1.69^a^^b^	14.10	24.58±1.86^a^^b^	14.28	23.72±1.46^a^^b^	23.20	39.02±1.05^b^	16.20	0.029	0.6364
**ALP (UL**^**-1**^**)**^**7**^	17.21±1.23^a^	16.08	30.09±1.78^b^	9.59	32.99±1.07^b^	16.00	33.30±1.9^b^	6.38	46.85±1.98^c^	7.35	0.0001	0.8594
GPT (UL^-1^)^8^	156.8±8.42	22.87	163.6±6.84	23.09	166.6±7.00	25.25	173.3±9.40	21.90	176.0±6.66	9.95	0.427	0.3391
GOT (UL^-1^)^9^	2.97±0.18	16.56	3.17±0.19	16.42	3.12±0.22	18.87	3.47±0.23	17.50	3.43±0.20	15.48	0.393	0.3518

^1^ Experimental diet abbreviations: 0% PO (100% FO, 0% PO), 25% PO (75% FO, 25% PO), 50% PO (50% FO, 50% PO), 75% PO (25% FO, 75% PO), 100% PO (0% FO, 100% PO).

^2^Total cholesterol;

^3^Triglyceride;

^4^High-density lipoprotein cholesterol;

^5^Low-density lipoprotein cholesterol;

^6^Aspartate aminotransferase;

^7^Alkaline phosphatase;

^8^Glutamic-pyruvic transaminase;

^9^Glutamic- oxalacetic transaminase. Values are mean ± SEM (n = 15). Different superscripts indicate significant difference among different treatments (*P*<0.05).

‘Superscript a is significantly less than

b. Superscript b is significantly less than

c. Parameters highlighted have significant differences among groups. CV is coefficient of variation and r is correlation

### Serum total protein (TP), plasma lysozyme (LYZ) and respiratory burst (RB) activity

The results for non-specific immune response such as serum lysozyme (LYZ), total protein and respiratory burst (RB) are shown in Table 8. TP and RB increased significantly (p<0.05) with increasing PO levels. LYZ on the other had showed a trend of decreasing along increasing PO levels but was non-significant. TP ranged between 30.73 to 36.81 (mmol L^-1^), whiles LYZ and RB ranged between 159.7 to 184.8 (Uml^-1^) and 12.10 to 16.18 (540nm) respectively.

**Table 8:** Effects of dietary PO inclusion on serum lysozyme activity, total protein and respiratory burst of *Oreochromis niloticus* fed experimental diets.

	Dietary Palm oil (PO) replacement level (%)^1^											
Parameters	0% PO	CV	25% PO	CV	50% PO	CV	75% PO	CV	100% PO	CV	P value	r
Lys (Uml^-1^)^2^	184.8±13.92	19.93	172.0±14.61	22.48	1**5**9.7±12.40	20.55	177.6±11.63	17.33	161.5±11.68	19.13	0.6086	0.2890
**TP (mmol L**^**-1**^**)**^**3**^	30.72±1.05^a^	7.66	35.32±1.19^a^^b^	7.58	36.81±1.85^b^	18.25	36.87±0.81^b^	4.92	35.10±1.05^a^^b^	6.72	0.014	0.6697
**RB (540nm)**^**4**^	12.10±0.40^a^	6.67	12.43±0.23^a^^b^	6.89	12.61±0.19^a^^b^	4.64	14.61±0.21^b^^c^	3.15	16.18±0.29^c^	3.27	0.0009	0.9063

^1^ Experimental diet abbreviations: 0% PO (100% FO, 0% PO), 25% PO (75% FO, 25% PO), 50% PO (50% FO, 50% PO), 75% PO (25% FO, 75% PO), 100% PO (0% FO, 100% PO).

^2^Lysozyme activity;

^3^Total protein;

^4^Respiratory burst. Values are mean ± SEM (n = 15). Different superscripts indicate significant difference among different treatments (*P*<0.05).

Superscript a is significantly less than

b. Superscript b is significantly less than

c. Parameters highlighted have significant differences among groups. CV is coefficient of variation and r is correlation.

### Effects of dietary palm oil levels on antioxidant capacity

The effects of dietary palm oil on antioxidant capacity are shown in Table 9. There was no significant difference in serum GSH-Px activity. Also, dietary PO inclusion did not significantly affect CAT and SOD. There was a significant decrease in serum MDA when fish were fed 100% PO. Mean MDA activity ranged between 2.70–5.12. Also, there was a significant decrease in the serum TAC as dietary PO levels increased. Fish fed diets containing 0% PO and 25% PO recorded significantly lower GST activity compared to fish fed 75% PO and 100% PO but was non-significantly lower than those fed 50% PO level. Group fed higher levels of dietary PO (75% and 100%) recorded significantly lower levels of serum GR activity compared to those fed 0% PO, 25% PO and 50% PO levels.

**Table 9:** Effects of dietary PO inclusion on antioxidants of Nile tilapia fed experimental diets for 8 weeks.

	Dietary Palm oil (PO) replacement level (%)^1^											
Parameters	0% PO	CV	25% PO	CV	50% PO	CV	75% PO	CV	100% PO	CV	P value	r
**GR(U/L)**^**2**^	383.5±17.86^b^	11.40	382.9±16.69^b^	10.68	365.7±27.45^b^	18.39	230.1±30^a^	18.42	202.2±18.78^a^	22.76	0.0001	0.8720
**TAC (U/L)**^**3**^	50.73±2.40^c^	11.63	33.61±2.33^b^	16.98	28.06±2.26^a^^b^	19.77	22.79±3.00^a^	12.27	20.00±0.70^a^	8.58	0.0001	0.9060
**MDA (nmol/ml)**^**4**^	5.12±0.32^b^	15.71	4.35±0.40^b^	22.80	4.14±0.26^a^^b^	15.39	4.04±0.50^a^^b^	20.42	2.70±0.16^a^	15.35	0.0014	0.7042
**GST (U/L)**^**5**^	277.6±25.92^a^	22.87	292.3±27.55^a^^b^	23.09	424.2±43.73^b^^c^	25.25	452.0±40.40^c^	21.90	479.8±19.49^c^	9.95	0.0002	0.7533
CAT (U/L)^6^	1.38±0.05	5.12	1.28±0.08	8.84	1.27±0.06	6.68	1.26±0.08	8.98	1.22±0.04	4.64	0.5460	0.6381
SOD (U/L)^7^	22.72±1.45	9.03	20.23±0.97	6.78	19.72±1.43	10.26	21.42±1.11	7.33	21.12±1.38	9.24	0.5657	0.6285
GSHpx (U/L)^8^	40.51±3.81	23.08	36.99±5.71	17.87	35.10±3.82	16.71	28.50±3.41	19.32	30.54±3.86	21.03	0.2862	0.4189

^1^ Experimental diet abbreviations: 0% PO (100% FO, 0% PO), 25% PO (75% FO, 25% PO), 50% PO (50% FO, 50% PO), 75% PO (25% FO, 75% PO), 100% PO (0% FO, 100% PO).

^2^Glutathione reductase;

^3^Total antioxidant capacity;

^4^Malondialdehyde;

^5^Glutathione s-transferase;

^6^Catalase (CAT);

^7^Superoxide dismutase;

^8^Glutathione peroxidase (GSH-Px). Values are mean ± SEM (n = 15). Different superscripts indicate significant difference among different treatments (*P<*0.05).

Superscript a is significantly less than

b. Superscript b is significantly less than

c. Parameters highlighted have significant differences among groups. CV is coefficient of variation and r is correlation.

### Mortality rate in *S*. *iniae* challenge

There was no difference (*P*>0.05) in mortality among groups post bacteria (*S*. *iniae*) challenge (Table 10). Mortality ranged between 36.33 to 63.33%.

**Table 10:** Effects of replacing FO with PO on post-challenge mortality of *Oreochromis niloticus* after infection with *Streptococcus iniae*.

	Dietary Palm oil (PO) replacement level (%)^1^										
0% PO	CV	25% PO	CV	50% PO	CV	75% PO	CV	100% PO	CV	p-value	r
36.67±3.33	15.75	43.33±6.66	26.65	60.00±5.77	16.67	40.00±10.00	43.43	63.33±6.66	18.23	0.06	0.6971

^1^ Experimental diet abbreviations: 0% PO (100% FO, 0% PO), 25% PO (75% FO, 25% PO), 50% PO (50% FO, 50% PO), 75% PO (25% FO, 75% PO), 100% PO (0% FO, 100% PO). Values are mean ± SEM (n = 3). CV is coefficient of variation and r is correlation.

## Discussion

Generally, the results of this study showed that PO inclusion had no negative impact on growth performance in *O*. *niloticus*. This indicates that PO could be used to replace FO in *O*. *niloticus* diets either partially or totally. The higher growth rate coupled with lower FCR recorded in fish fed 50% suggested that 50% PO inclusion could maintain normal physiological functions for *O*. *niloticus*. The results of this study was similar to previous studies for juvenile Nile tilapia [29], juveniles Mozambique tilapia (*O*. *mossambicus*) [30] and common carp [31] when dietary FO were substituted by vegetable oils at different levels. Although fish fed diets containing 25% PO performed quite poorly in terms of weight gain and specific growth rate, it was not significantly different from groups fed diets containing 0% PO, 75% PO and 100% PO. This is because tilapia, like most fresh water fish, are known to have a greater requirement for n-6 fatty acids predominantly found in vegetable oils as compared to n-3 fatty acids which are dominant in FO for proper growth [32]. This is also because when EFA requirements are met a significant proportion of dietary FO can be replaced by other lipid sources without any compromise in growth performance, feed intake as well as feed efficiency [33]. There was no significant difference in FCR among different treatments, which is in agreement to a previous study [31], indicating that fish had similar ability with respect to feed digestion and converted the diets into body tissue with same degree of efficiency [34]. The above results signify that using PO in place of FO in Nile tilapia has a great potential of increasing production since growth performance wouldn’t be compromised.

The effects of different dietary palm oil inclusion on fatty acid composition and lipid levels (liver and muscle) [17], a study directly related to this study. The continuous demand for aquatic products continue to rise due to the benefits (nutritional and health) obtained from the consumption of these products (Sargent et al. 2001). The entire body of fish is composed of approximately 40–60% skeletal muscle. Based on this premise, the effects of replacing fish oil with vegetable oils on nutritional and health benefits of the muscle is of great interest. The results indicate that feeding *O*. *niloticus* with higher levels of palm in place of fish oil could be compromised. This is evident from the fact that compared to the control, groups fed 75% PO and 100% PO recorded significantly low amounts of EPA and DHA. This is in agreement to previous studies by [31] when fish oil was replaced with rapeseed oil in diets of black carp fingerlings. Another indicator of nutritional composition of diets (fish) is the n-3: n-6 composition. Diets with high amounts of n-3 PUFA as well as lower levels of n-6 PUFA are essential to human health since they such diets contain higher n-3: n-6 ratios. The results of n-3 PUFA, n-6 PUFA and n-3: n-6 ratios imply feeding *O*.*niloticus* with PO in place could negatively affect the nutritional status of the final product. This is because groups fed 75% PO and 100% PO recorded the least n-3: n-6 ratio and is in agreement to previous study [28] which reported that feeding *O*. *niloticus* with coconut oil reduces n-3: n-6 ratios.

It has been reported that an increase in liver ALP as well as increase in plasma ALP is an indicator of damage to the parenchyma in the liver [35]. In this study, feeding *O*. *niloticus* with varying levels of PO in place of FO increased ALP activity with fish fed 100% PO recording the highest value (Table 6). This indicates feeding fish (*O*. *niloticus*) with PO could affect the liver negatively although this study had no data on liver ALP. Fish fed 0% PO diet might have recorded the least ALP activity value due to a decrease in trans-phosphorylation activity [36]. The highest level of AST was recorded when *O*. *niloticus* were fed diet containing 100% PO as the sole lipid source and was significantly higher than those fed diets with 0% PO. This is an indication that feeding *O*. *niloticus* on PO diets could influence the liver negatively even though [26] documented that these parameters were not specifically for liver malfunctioning but to access active hepatic damage. However, this study was in contrast to previous study where PO inclusion reduced AST levels [34]. This study recorded triacylglycerol (TAG) values increasing along increasing PO levels with fish fed diets containing 50%, 75% and 100% PO recording values significantly higher than those fed diets containing 0% PO. The results confirmed that vegetable lipid enhance TAG secretion and production, whereas fish oil decrease it [30]. Although TAG levels increased with PO inclusion levels, fish fed 100% PO recorded the least HDL-C/LDL-C ratio which could imply feeding fish on such diet could strengthen their ability to use lipid effectively as a reduction in the HDL-C/LDL-C ratio of fish depicts the utilization ability of lipids is strengthened [37]. The results of this study revealed that PO inclusion increased LDL-C and decreased HDL-C. Fish fed diets containing 75% PO and 100% PO recorded LDL-C values significantly higher than those fed the CTRL diet. This was an indication of good transport of cholesterol from the liver to tissues and metabolic activities [37]. Also, decrease in HDL-C along increasing PO inclusion served as a good indicator for clean and healthy system needed for prevention of some diseases. The increase in LDL-C and decrease in HDL-C along increase in PO inclusion recorded in this study was in agreement to previous study [38] and indicates cholesterol levels were increased as PO oil levels increased in the diet. This is in agreement to previous study [39].

Although the defence capability of the mucosal surface was reduced by the inclusion of PO due to the reduction in lysozyme activity, the lysozyme activity not being significantly altered by PO inclusion suggested that replacing FO with PO in diets of *O*. *niloticus* did not deplete disease resistance. This is in agreement to previous studies, which reported that serum lysozyme activity was not affected by FO substitution with VOs in Eurasian perch [40], Nile tilapia [41] and Gilthead sea bream [42]. On the contrary, other studies suggested that replacing FO with VOs had significant effects on serum lysozyme activity in juvenile dark barbel catfish [43]. These differences in results might have arisen owing to the differences in dietary fatty acid composition and the ratio of n-6/n-3 fatty acids of diets which are known to have effects on immunity and disease resistance in fish [42, 44]. Respiratory burst (RB) and total protein (TP) were significantly increased by dietary PO levels signifying replacing FO with PO does not compromise the health of fish (tilapia).

There was a decrease in the antioxidant activities of GSHpx in this study as dietary PO levels increased. This signifies that feeding tilapia with PO decreases peroxidative damage through removal of excessive ROS. This study recorded a decline in SOD and CAT activities in the serum of *O*. *niloticus* when fed diets with different PO. This is in agreement to the study of [31] in which replacing FO with rapeseed oil resulted in decline of SOD. The decline in SOD and CAT might be attributed in the changes in the dietary n-3 HUFA concentration [31]. There was a significant decrease in MDA as PO levels increased indicating a reduced susceptibility to fatty acid peroxidation. This could also signify that supplementing diets with PO prevents the accumulation of MDA effectively therefore lipid peroxidation was suppressed by PO. There were significantly higher mean GST values in fish fed diets containing more than 50% PO in this present study which suggest PO improved the cytoprotective role of Nile tilapia. A significant reduction in TAC levels was recorded as PO levels increased and is in agreement to the study by [45] in which total replacement of FO with vegetable oil led to a reduced TAC activity. This study records higher levels of GR, GSHpx, MDA, TAC and CAT in fish fed higher levels of FO, this could attributed to the higher content of n-3 fatty acids in FO which could prevent the suppression of antioxidant enzyme activities [45].

Replacement of fish oil with palm oil affected the amylase activity in AI, MI as well as PI (Table 4). The least amylase activity was recorded in the PI (22–29) compared to AI and MI (25–32 and 24–32, respectively) in all experimental groups. This is probably because, most part of nutrient digestion in fish generally takes place in the anterior intestine and, to a lesser extent, in the mid intestine; the posterior intestine usually has lower digestive enzyme activity [46]. In the AI, fish fed 50% PO recorded the highest level of amylase activity and was significantly higher than those fed 25% PO, 75% PO and 100% PO levels. Similarly the activity of amylase in both MI and PI were stimulated in fish fed 50% PO and followed same trend as in AI. Lipase activity in all sections (AI, MI and PI) generally increased as PO levels increased in experimental diets. Lipase activity was affected by the replacement of fish oil with palm oil but recorded a trend of increasing activity with increasing PO level. This is contrary to previous study by [19] which showed that substituting fish oil with canola oil in diet of yellow tail kingfish (*Seriola lalandi*) did not affect lipase activity. Lipase activity in AI in fish fed diets containing 25%, 50%, 75% and 100% PO were higher compared to the values in MI and PI. This phenomenon is considered artificial and might have been caused by a possible drag of secreted mucus to that part of the digestive tract [46]. The study also revealed a strong positive correlation between dietary palm oil inclusion and lipase activity in *O*. *niloticus*, which indicates that *O*. *niloticus* can digest different palm oil levels, and utilized them as an energy source. In this study, fish fed 50% PO had the highest protease enzyme activity in AI, MI and DI. There existed a correlation between enzyme activity and growth rate implying digestive enzyme play significant role in the growth of Nile tilapia.

Replacement of FO with PO did not significantly influence on the resistance ability of *O*. *niloticus*. This was in agreement to previous study where dietary lipid levels had no effects on the resistance of Nile tilapia to *S*. *iniae* infection [47]. Contrary to our study, different lipid sources significantly influenced the resistance ability in channel catfish, *Ictalurus punctatus* [48]. These differences could be attributed to the differences in fish species, sizes, dietary lipid sources and/or levels, feeding duration as well as environmental factors [35]. The difference in diet composition (fatty acids) used in these studies could also be an underlining factor for these discrepancies as fish immunity as well as disease resistance can be affected due to the changes in dietary fatty acid composition and the ratio of n-6/n-3 fatty acids [44, 49]. The non-significance difference in the resistance to *S*. *iniae* is important because *S*. *iniae* is known to cause serious mortalities of more than 40%. The above results shows that replacing FO with PO, although wouldn’t improve resistance wouldn’t either compromise the ability of Nile tilapia to fight *S*. *iniae*.

## Conclusion

In conclusion, this study demonstrated that FO could be replaced with PO without distinct compromise in growth performance as well as feed utilization in Nile tilapia. However, nutritional and health benefits of feeding *O*. *niloticus* diets with higher levels of PO (75% PO and 100% PO) is reduced due to the decrease in EPA and ARA with corresponding increase in n-3:n-6 ratios. There was an increase in AST and ALP which suggested that supplementing fish diet with PO could lead to liver damage. Compared to FO, PO improved innate immune parameters (Total protein and Respiratory burst) of juvenile Nile tilapia. With the exception of GST which increased significantly with increasing dietary PO, replacing FO with PO significantly reduced GR, TAC and MDA. Finally, there was no difference in resistance ability of juvenile tilapia when challenged with *S*. *iniae*.
